# Not Only in Sensorimotor Network: Local and Distant Cerebral Inherent Activity of Chronic Ankle Instability—A Resting-State fMRI Study

**DOI:** 10.3389/fnins.2022.835538

**Published:** 2022-02-07

**Authors:** Yiyuan Shen, Weiwei Wang, Yin Wang, Liqin Yang, Chengjie Yuan, Yang Yang, Fei Wu, Junlong Wang, Yan Deng, Xu Wang, Hanqiu Liu

**Affiliations:** ^1^Department of Radiology, Huashan Hospital, Fudan University, Shanghai, China; ^2^Department of Radiology, Eye & ENT Hospital, Fudan University, Shanghai, China; ^3^Institute of Functional and Molecular Medical Imaging, Fudan University, Shanghai, China; ^4^Department of Orthopedic, Huashan Hospital, Fudan University, Shanghai, China; ^5^Department of Radiology, Zhongshan Hospital, Fudan University, Shanghai, China

**Keywords:** chronic ankle instability, resting-state fMRI, amplitude of low-frequency fluctuation, regional homogeneity, functional connectivity

## Abstract

**Background:**

Increasing evidence has proved that chronic ankle instability (CAI) is highly related to the central nervous system (CNS). However, it is still unclear about the inherent cerebral activity among the CAI patients.

**Purpose:**

To investigate the differences of intrinsic functional cerebral activity between the CAI patients and healthy controls (HCs) and further explore its correlation with clinical measurement in CAI patients.

**Materials and Methods:**

A total of 25 CAI patients and 39 HCs were enrolled in this study. Resting-state functional magnetic resonance imaging (rs-fMRI) was used to detect spontaneous cerebral activity. The metrics of amplitude of low-frequency fluctuation (ALFF), fractional ALFF (fALFF), and regional homogeneity (ReHo) of the two groups were compared by two-sample *t*-test. The brain regions that demonstrated altered functional metrics were selected as the regions of interest (ROIs). The functional connectivity (FC) was analyzed based on the ROIs. The Spearman correlation was calculated between rs-fMRI metrics and clinical scale scores.

**Results:**

Compared with HCs, CAI patients showed higher ALFF and ReHo values in the right postcentral gyrus, the right precentral gyrus, and the right middle frontal gyrus, while lower fALFF values in the orbital-frontal cortex (OFC, *p* < 0.01 after correction). Increasing FC between the right precentral gyrus and the right postcentral gyrus while decreasing FC between the right precentral gyrus and the anterior cingulum cortex (ACC), the right middle frontal gyrus and the left middle temporal gyrus, and the OFC and left inferior parietal lobule (IPL) was observed. In addition, in the CAI group, the ReHo value negatively correlated with the Cumberland Ankle Instability Tool score in the right middle frontal gyrus (*r* = −0.52, *p* = 0.007).

**Conclusion:**

The CAI patients exhibited enhanced and more coherent regional inherent neuronal activity within the sensorimotor network while lower regional inherent activity in pain/emotion modulation related region. In addition, the information exchanges were stronger within the sensorimotor network while weaker between distant interhemispheric regions. Besides, the increased inherent activity in the right middle frontal gyrus was related to clinical severity. These findings may provide insights into the pathophysiological alteration in CNS among CAI patients.

## Introduction

Chronic ankle instability (CAI), usually developing from the acute ankle sprain, is one of the most prevalent musculoskeletal injuries (Gribble et al., 2016). CAI is a generic term characterized by a group of symptoms referring to recurrent sprain, episodes of ankle joint “giving way,” pain, swelling, and decreased function (Delahunt et al., 2010; Gribble et al., 2014). These residual symptoms can last for decades, impacting physical activity levels and sacrificing the health-related quality of life (Houston et al., 2015). Moreover, repetitive trauma can degenerate joint cartilage, leading to early onset of post-traumatic osteoarthritis (Lee et al., 2015), which lacks effective treatment (Yeo et al., 2021). Nowadays, conservative rehabilitation and surgery after ineffectual conservative management are the primary treatment for CAI (Yeo et al., 2016; Kim et al., 2019). However, some patients fail to recover or return to sports as usual (Doherty et al., 2017; Aicale and Maffulli, 2020). Hence, it will be beneficial to explore the pathophysiological alterations underlying CAI to improve its interventions.

According to the classic Hertel model (Hertel, 2002) of CAI, the impairment of neuromuscular-recruitment pattern is one of the pathophysiological factors. As is known, the central nervous system (CNS) can adapt to the periphery-induced stimuli. Neuroplasticity refers to the ability of the brain to reorganize synaptic connections, functional networks, or morphological structures in response to stimuli like peripheral injury (Kapreli et al., 2009; Grooms et al., 2015). During the past decade, much effort has been made to investigate the neuroplasticity of CAI patients. Some studies found that CAI patients had decreased corticomotor excitability in fibularis longus (FL), tibialis anterior, and soleus, which supported the existence of supraspinal alteration (Hass et al., 2010; McLeod et al., 2015; Terada et al., 2016, 2020; Nanbancha et al., 2019). Recently, Kosik et al. (2017) has found that CAI patients had reduced corticomotor cortex output representation of FL, indicating the FL corresponding motor cortical cells were recruited by surrounding areas. These studies revealed the reorganization of the corticomotor area. Therefore, CAI is not only a peripheral problem related to ligaments and muscles but also a problem related to the alteration of CNS. Whereas, most studies of the CAI-induced neuroplasticity were based on transcranial magnetic stimulation (TMS), which reflected patients’ response after externally given stimuli and was limited by the restricted target area of TMS, mainly focusing on the motor-related cerebral changes. However, CAI involves multiple aspects like motor deficits and pain management, and CNS alterations may not be confined to sensorimotor networks. Nevertheless, it is still unclear whether the inherent brain activity involving these factors of CAI patients is altered.

In this study, resting-state fMRI (rs-fMRI) was used to observe the intrinsic cerebral activity, which has been widely applied in various neurological or psychiatric disorders (Liu et al., 2019; Xu et al., 2020a,b; Lin et al., 2021). We utilized data-driven algorithms, including the amplitude of low-frequency fluctuation (ALFF) (Zang et al., 2007), fractional ALFF (fALFF) (Zou et al., 2008), regional homogeneity (ReHo) (Zang et al., 2004), and functional connectivity (FC) (Hampson et al., 2002), which complement each other on different aspects, to reflect the intrinsic cerebral activity. ALFF, fALFF, and ReHo are local metrics. ALFF highlights the intensity of regional neuronal activity, while fALFF reflects the contribution of the low-frequency band for the whole frequency range and is considered less affected by physiological noise. ReHo reveals the similarity of regional neuronal activity from the spatial perspective (Zang et al., 2004, 2007; Zou et al., 2008). On the other hand, FC can provide information about extensive functionally connected regions. Therefore, combining the above metrics can provide a comprehensive evaluation of spontaneous cerebral activity on a whole-brain scale, facilitating understanding of the reorganization in brain function in CAI patients and contributing to further understanding of the pathophysiological changes of CAI patients.

Hence, the aim of this work was to investigate the alteration in intrinsic cerebral activity in CAI patients compared with healthy controls (HCs) by rs-fMRI and explore the relationship between functional metrics and clinical measurement. We hypothesized that CAI patients would differ from HCs in resting-state metrics, and the altered functional metrics will be associated with clinical symptoms.

## Materials and Methods

### Participants

A total of 25 patients with right side CAI (26.24 ± 5.33 years) were enrolled in the patient group between September 2019 and April 2021 from the Department of Orthopedics of our hospital. Since most of the collected patients were right-side suffered, only right-side CAI patients were included in this study, allowing aggregation of the cerebral activity data without interference from unique unilateral brain changes that might be confounded if the cohort had mixed left and right CAIs. A total of 39 HCs (27.79 ± 4.69 years) were recruited in this study from the community. The Chinese translation of Edinburgh Handedness Inventory was used to assess the handedness (Yang et al., 2018). All the participants were right-handed. The CAI patients underwent cross-sectional evaluation of clinical assessments and MR scanning. For the patient group, the inclusion criteria consisted of Gribble et al. (2014): (i) A history of at least one significant ankle sprain leading to at least 3 days of immobilization and/or non-weight bearing, and the initial sprain must have occurred at least 12 months before the study enrollment. The most recent injury must have occurred more than 3 months before the study enrollment. (ii) A history of the previously injured ankle joint at least two episodes of “giving way” in the 6 months before the study enrollment, and/or recurrent sprain and/or “feelings of instability.” (iii) Cumberland Ankle Instability Tool (CAIT) score less than 24.

Exclusion criteria for both groups were as follows: (i) A history of previous surgeries to the musculoskeletal structures (i.e., bones, joint structures, and nerves) or a fracture requiring realignment in either lower extremity. (ii) Acute injury to the musculoskeletal structures of other lower extremity joints in the previous 3 months, which impacted joint integrity and function (i.e., sprains and fractures), resulting in at least one interrupted day of desired physical activity. (iii) A history of bilateral ankle sprains. (iv) CNS diseases, muscular diseases, and other conditions that may have influences on ankle movement. (v) Confirmed or suspected history of cardiopulmonary failure. (vi) Psychiatric disorders. (vii) Concurrent and contraindications to an investigation by MRI. All participants volunteered to undergo the rs-fMRI scanning.

The current study was conducted in accordance with the Declaration of Helsinki, and all study procedures were carried out with adequate understanding and the written consent of the participants. Formal approval from the Huashan Hospital Institutional Review Board was obtained before study initiation.

### Clinical Assessments

The CAIT, American Orthopedic Foot and Ankle Society (AOFAS), and Karlsson–Peterson Ankle Function Score (KPAFS) are frequently used scales to evaluate the ankle joint from different perspectives. The CAIT is disease-specific, while AOFAS and KPAFS are body region-specific. Combining the three scales will help provide a more comprehensive assessment of the ankle joint.

Cumberland Ankle Instability Tool (Hiller et al., 2006): CAIT is usually used to evaluate the subjective feelings of the ankle in daily activities such as walking, running, jumping, and going down the stairs. There are nine questions in this scale, and the total score is 30. According to the International Ankle Consortium (Gribble et al., 2014), a score less than 24 is used to differentiate the unstable ankles from the healthy ones. In addition, the lower the score, the worse the ankle stability. Therefore, CAIT can not only distinguish whether the joint is stable, but also define the severity of symptoms.

American Orthopedic Foot and Ankle Society Score (Kitaoka et al., 1994): the AOFAS scale is a clinician-based scale to assess the ankle and foot disorders from different parts comprising pain, function, and alignment. The total score ranges from 0 to 100. The score of AOFAS ranging 0–49, 50–74, 75–89, and 90–100 indicates poor, fair, good, and excellent, respectively.

Karlsson–Peterson Ankle Function Score (Cao et al., 2018): the KPAFS is a method of evaluating function by examining the stability of the ankle joint, pain, swelling, numbness, activities at work or during sports, the ability to climb stairs, running ability, and the use of ankle support aids. The total score ranges from 0 to 100. The score of KPAFS ranging 0–59, 60–74, 75–84, and 85–100 indicates poor, fair, good, and excellent, respectively.

### Data Acquisition

All imaging data were obtained with a 3.0 T scanner (MR750, GE Healthcare, Milwaukee, WI, United States) equipped with a 32-channel head coil. The participants’ heads were fastened by cushions between both sides of their head and coil to minimize the head movements. During the scanning, the participants were required to relax their minds and not think about anything, keeping awake with their eyes closed. Each participant received one functional scan. The rs-fMRI data were measured with an echo-planar imaging sequence (TR/TE = 2,000/30 ms, flip angle = 90°, FOV = 220 mm × 220 mm, 43 axial slices, acquisition matrix = 64 × 64, voxel size = 3.4 mm × 3.4 mm × 3.2 mm, interslice space = 0 mm). Structural imaging data were acquired with a 3D T1-weighted fast spoiled gradient-recalled echo sequence (TR/TE = 8.16/3.18 ms, inversion time = 450 ms, flip angle = 8°, FOV = 256 mm × 256 mm, acquisition matrix = 256 × 256, spatial resolution = 1 mm × 1 mm × 1 mm, interslice space = 0 mm).

### Data Preprocessing

The rs-fMRI data were preprocessed by Data Processing and Analysis for Brain Imaging (DPABI) (Yan et al., 2016) and Statistical Parametric Mapping (SPM12) (Biswal et al., 2010) on MATLAB 2019b (MathWorks, Natick, MA, United States). The steps are as follows: (i) Remove the first 10 volumes to ensure magnetization stabilization. (ii) Slice timing was conducted to the middle slice to eliminate the variances due to the different acquisition times. (iii) Realignment was performed using a six-parameter rigid-body spatial transformation to compensate for head-movement artifacts. Subjects with head-movement >3 mm of translation and >3° of rotation would be excluded, and no patient was excluded. (iv) The functional images were co-registered to the high-resolution 3D-T1 structural images. In this phase, structural images were normalized to Montreal Neurological Institute (MNI) space by non-linear warping based on Diffeomorphic Anatomical Registration Through Exponentiated Lie Algebra (DARTEL). Then the functional images were spatially normalized to the MNI space using the parameters achieved from the normalization of structural images and simultaneously resampled into 3-mm isotropic voxels. (v) Nuisance covariates were regressed out, including the Friston 24-motion parameter model (six head-motion parameters, six head-motion parameters one time point before, and the 12 corresponding squared items), global mean, white matter, and cerebrospinal fluid signals. (vi) Linear detrending and band-pass filtering at 0.01–0.08 Hz were carried out to reduce low-frequency drift and high-frequency physiological noise.

### Amplitude of Low-Frequency Fluctuation, Fractional Amplitude of Low-Frequency Fluctuation, and Regional Homogeneity Computation

A 6-mm full-width at half-maximum (FWHM) Gaussian kernel was used to smooth the normalized functional images for ALFF computation. Then a fast Fourier transform was used to convert the filtered time series to a frequency domain to obtain the power spectrum. The square root of the power spectrum was computed and averaged within each voxel, which was the ALFF. The term fALFF was the division of ALFF within the specified frequency band (0.01–0.08 Hz) by the entire frequency range (Zang et al., 2007; Zou et al., 2008).

For ReHo, Kendall’s coefficient of concordance of the time courses within each voxel and its 26 neighboring voxels was calculated. Then smoothing was performed with a 6 mm FWHM Gaussian kernel (Zang et al., 2004).

To obtain an approximately normal distribution, we conducted a Fisher’s Z transformation for the ALFF, fALFF, and ReHo. Ultimately, the Z-standardized map of each participant was used for the following statistical analysis.

### Functional Connectivity Analysis

Functional connectivity was calculated based on the results of ALFF, fALFF, and ReHo analysis to investigate the connectivity between regions of interest (ROIs) and the rest of the brain. The regions with aberrant intrinsic cerebral activity were selected as ROIs: including the right precentral gyrus, the right postcentral gyrus, the right middle frontal gyrus, and the right orbital-frontal cortex (OFC). The mean time series of each ROI was extracted, and Pearson’s correlation was performed between the time series of each ROI and each voxel. Finally, a Fisher’s Z transformation was performed to improve the normality.

### Statistical Analysis

The intergroup comparison of demographic and clinical data was performed by SPSS (version 20, Chicago, IL, United States). A two-sample *t*-test was used to assess the continuous variables, including age, height, weight, and clinical measurements. Chi-square test was used to test the significant gender difference. *p*-Value < 0.05 was considered statistically significant.

The functional metrics were compared between the CAI and HCs using SPM12 with a general linear model. For functional metrics, gender, age, height, and weight were regarded as covariates. Gaussian Random Field theory (GRF) was applied to multiple comparison correction, with the statistical threshold of *p* < 0.01 and cluster *p* < 0.05 (one-tailed) (Friston et al., 1994). The regions with a significant difference in functional metrics were regarded as ROIs. The mean value of ALFF, fALFF, and FC in ROIs was extracted in the CAI group, and the Spearman correlation test was performed to explore the functional metrics with clinical assessment by SPSS (version 20, Chicago, IL, United States). The calculations were performed after controlling the influences of age, gender, height, and weight. *p*-Value < 0.05 was considered statistically significant.

## Results

### Demographic and Clinical Characteristics

The demographic and clinical characteristics were shown in Table 1. There were no significant differences in age, gender, height, and weight between CAI patients and HCs. The score for CAIT, AOFAS, and KPAFS of CAI patients was 12.68 ± 4.96, 62.56 ± 11.72, and 50.88 ± 15.19, respectively, which were significantly lower than HCs.

**TABLE 1:** Demographic and clinical characteristics of participants.

	CAI	Control	*t*/χ^2^	*p-*Value
*n* (male/female)	25 (10/15)	39 (19/20)	0.47	0.49^a^
Age (year), mean (SD)	26.24 (5.33)	27.79 (4.69)	1.23	0.22^b^
Height (cm), mean (SD)	169.16 (6.57)	168.74 (9.25)	−0.21	0.83^b^
Weight (kg), mean (SD)	62.76 (15.00)	64.91 (14.19)	0.58	0.57^b^
CAIT, mean (SD)	12.68 (4.96)	29.59 (0.88)	16.86	<0.0001^b^
AOFAS, mean (SD)	62.56 (11.72)	99.74 (1.60)	15.78	<0.0001^b^
KPAFS, mean (SD)	50.88 (15.19)	98.33 (2.89)	15.44	<0.0001^b^

*^a^Chi-square test. ^b^Independent t-test. SD, standard deviation; CAI, chronic ankle instability; CAIT, Cumberland Ankle Instability Tool; AOFAS, American Orthopedic Foot and Ankle Society Score; KPAFS, Karlsson–Peterson Ankle Function Score.*

### Amplitude of Low-Frequency Fluctuation, Fractional Amplitude of Low-Frequency Fluctuation, and Regional Homogeneity Analysis

**FIGURE 1:**
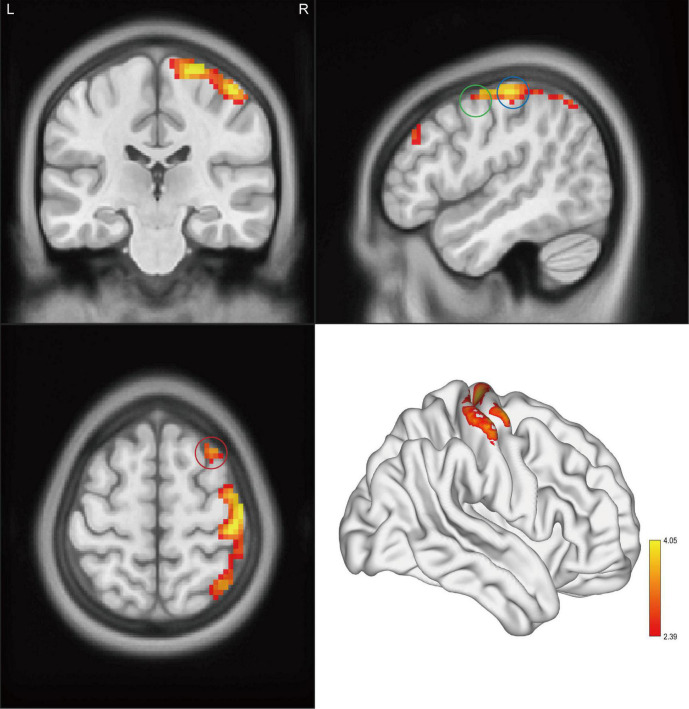
Clusters showed a significant difference in ALFF between CAI patients and HCs. The color bar indicated the *t*-value from two-sample *t*-test. The circles indicated the corresponding brain regions. The green circle: the right precentral gyrus; the blue circle: the right postcentral gyrus; and the red circle: the right middle frontal gyrus.

**FIGURE 2:**
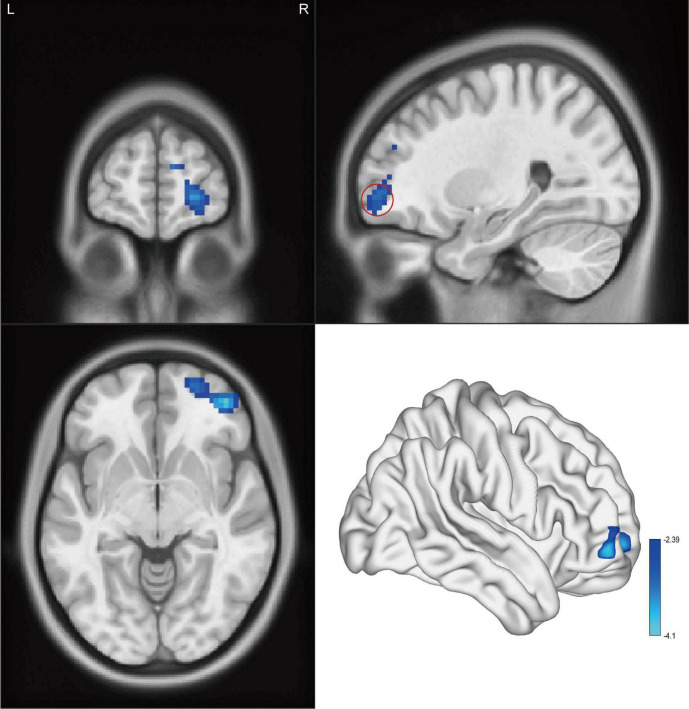
Cluster showed a significant difference in fALFF between CAI patients and HCs. The color bar indicated the *t*-value from two-sample *t*-test. The red circle indicated the orbital-frontal cortex.

**FIGURE 3:**
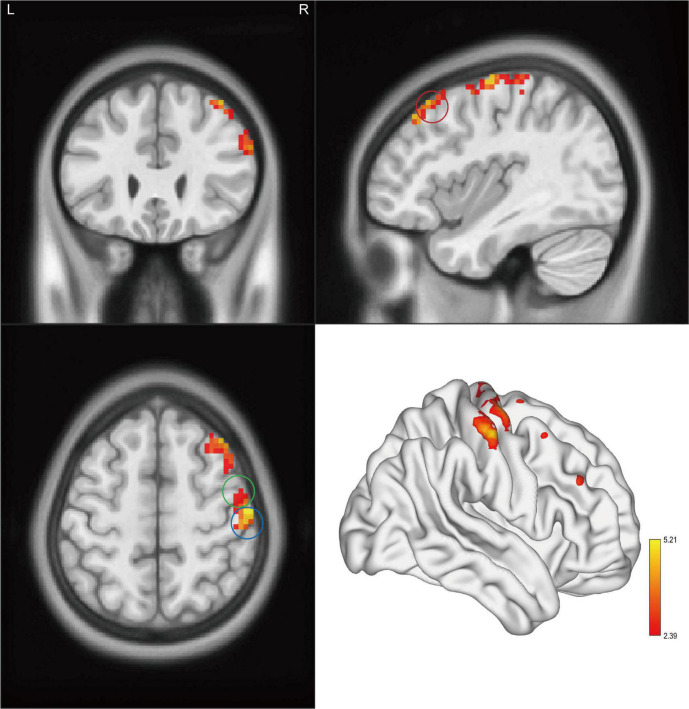
Clusters showed a significant difference in ReHo between CAI patients and HCs. The color bar indicated the *t*-value from two-sample *t*-test. The circles indicated the corresponding brain regions. The green circle: the right precentral gyrus; the blue circle: the right postcentral gyrus; and the red circle: the right middle frontal gyrus.

Significant intergroup differences in ALFF, fALFF, and ReHo were shown in Figures 1–3 and Tables 2–4. Compared with HCs, CAI patients demonstrated higher ALFF values in the right postcentral gyrus, extending to the right precentral gyrus and right middle frontal gyrus. For fALFF values, CAI patients had a lower level in the right middle frontal gyrus with its orbital part, extending to the right superior frontal gyrus with its medial part. Besides, ReHo values increased in the right precentral gyrus, right postcentral gyrus, and right middle frontal gyrus.

**TABLE 2:** Brain regions showing significant ALFF differences between CAI patients and HCs.

Region	BA	Cluster size	*t*-value for peak voxels	MNI coordinates, mm		
				*x*	*y*	*z*
PTs > HCs						
Postcentral_R	3	558	4.05	48	−24	60

*BA, Brodmann area; MNI, Montreal Neurological Institute; PTs, CAI patients; HCs, healthy controls; Postcentral_R, the right postcentral gyrus.*

**TABLE 3:** Brain regions showing significant fALFF differences between CAI patients and HCs.

Region	BA	Cluster size	*t*-value for peak voxels	MNI coordinates, mm		
				*x*	*y*	*z*
PTs < HCs						
Frontal_Mid_Orb_R	46	295	−4.10	42	51	−6

*BA, Brodmann area; MNI, Montreal Neurological Institute; PTs, CAI patients; HCs, healthy controls; Frontal_Mid_Orb_R, the right middle frontal gyrus, orbital part.*

**TABLE 4:** Brain regions showing significant ReHo differences between CAI patients and healthy controls.

Region	BA	Cluster size	*t*-value for peak voxels	MNI coordinates, mm		
				*x*	*y*	*z*
PTs > HCs						
Precentral_R	6	322	5.21	42	−9	66
Frontal_Mid_R	9	196	4.33	39	27	54

*BA, Brodmann area; MNI, Montreal Neurological Institute; PTs, CAI patients; HCs, healthy controls; Precentral_R, the right precentral gyrus; Frontal_Mid_R, the right middle frontal gyrus.*

### Functional Connectivity Analysis

**FIGURE 4:**
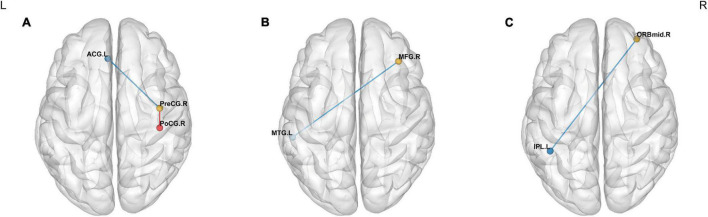
Intergroup comparison of FC between the CAI patients and HCs. The blue lines: decreased FC; the red line: increased FC. Compared with the HCs, the CAI group showed **(A)** decreased FC between the right precentral gyrus and the left ACC and increased FC between the right precentral gyrus and the right postcentral gyrus. **(B)** Decreased FC between the right middle frontal gyrus and left middle temporal gyrus. **(C)** Decreased FC between the OFC and the left IPL. ACG.L, the left anterior cingulate gyrus; PreCG.R, the right precentral gyrus; PoCG.R, the right postcentral gyrus; MFG.R, the right middle frontal gyrus; MTG.L, the left middle temporal gyrus; ORBmid.R, the orbital part of the right middle frontal gyrus; IPL.L, the left inferior parietal lobule.

Significant intergroup differences in FC were shown in Figure 4 and Table 5. Compared with the HCs, the CAI group showed increased FC between the right precentral gyrus and the right postcentral gyrus while decreased FC between the right precentral gyrus and the anterior cingulum cortex (ACC), the right middle frontal gyrus and the left middle temporal gyrus, and the OFC and left inferior parietal lobule (IPL).

**TABLE 5:** Brain regions showing significant FC differences between CAI patients and healthy controls.

Region	BA	Cluster size	*t*-value for peak voxels	MNI coordinates, mm		
				*x*	*y*	*z*
Seed1: Precentral_R						
Cingulum_Ant_L	32	82	−3.68	0	45	9
Postcentral_R	3	113	4.27	60	−18	48
Seed2: Frontal_Mid_R						
Temporal_Mid_L	21	73	−3.96	−60	−18	−9
Seed 3: Frontal_Mid_Orb_R						
Parietal_Inf_L	40	80	−4.53	−54	−51	36

*FC, functional connectivity; BA, Brodmann area; MNI, Montreal Neurological Institute; Precentral_R, the right precentral gyrus; Cingulum_Ant_L, the left anterior cingulate gyrus; Postcentral_R, the right postcentral gyrus; Frontal_Mid_R, the right middle frontal gyrus; Temporal_Mid_L, the left middle temporal gyrus; Frontal_Mid_Orb_R, the right middle frontal gyrus, orbital part; Parietal_Inf_L, the left inferior parietal lobule.*

### Correlations With Clinical Parameters

**FIGURE 5:**
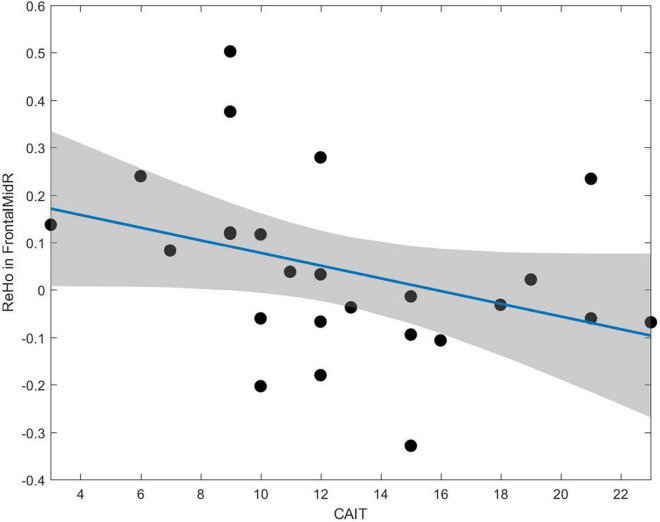
A negative correlation between the CAIT and ReHo value can be observed in the middle frontal gyrus (*r* = –0.52, *p* = 0.007).

The correlation between altered rs-fMRI parameters and clinical assessment was illustrated in Figure 5. The ReHo value negatively correlated with the CAIT score in the right middle frontal gyrus (*r* = −0.52, *p* = 0.007).

## Discussion

In the present study, we found differences in ALFF, fALFF, ReHo, and FC in CAI patients compared with HCs by rs-fMRI and explored their correlation with clinical parameters. The current results suggested that altered regional and extensive cerebral activities happened in the sensorimotor network and pain-related regions among CAI patients, which proved the neuroplasticity of inherent functional activity in CAI patients. To the best of our knowledge, this is the first study using rs-fMRI to investigate the inherent cerebral activity of CAI patients.

In this study, we found that the CAI patients showed increased ALFF and ReHo in the right postcentral gyrus, the precentral gyrus, and the middle frontal gyrus. These regions constitute the sensorimotor network (Wei et al., 2020). The postcentral gyrus (BA 3) is a part of the primary somatosensory cortex (S1), receiving the proprioceptive input (Srinivasan et al., 2020). The precentral gyrus (BA 4) belongs to the primary motor area (M1), participating in motor execution (Paul et al., 2021). The middle frontal gyrus, involving BA 6, 8, and 9, constitutes the premotor cortex and is engaged in the planning and preparing movements (Matsuo et al., 2021). It is well known that the completion of the movement is initiated by signals from the cerebral cortex, which reach the skeletal muscle motor endplate through outgoing fiber. The process involves motor plan generation, coordinated movement control, and corrective feedback. Notably, our results revealed that the ipsilateral sensorimotor network was more activated in CAI patients, probably due to the compensation of the unaffected ankle (left ankle). Some studies have found that CAI patients transformed their gait and posture to compensate for the injured ankle (Levin et al., 2015; Ziabari et al., 2021), which implied reassignment of cerebral function. Under physiological conditions, the two hemispheres are mutually inhibited, but the inhibition can be upset in certain circumstances, and cerebral function can be redistributed (Zhang J. et al., 2021). Hence, the increased functional cerebral metrics in the right sensorimotor network may be a compensatory strategy to maintain the balance of the whole body. When one ankle joint is injured, the ipsilateral brain may facilitate the uninvolved ankle to perform a greater role in keeping balance.

Meanwhile, we found that in the right middle frontal gyrus, the ReHo value was negatively correlated with CAIT, which was in line with our compensatory hypothesis. As mentioned before, this region plays a vital role in motor planning. In other words, the worse the ankle function, the more the brain activated the ipsilateral premotor area to innervate the unaffected ankle to compensate for the affected one. Nevertheless, motor control of the lower extremities is usually attended bilaterally (Grooms et al., 2017). Thus, the current results indicated that the integration of bilateral ankle training should be considered during rehabilitation to enhance coordination of bilateral lower extremity movements and avoid functional or strength asymmetry.

In addition, we found a reduction of fALFF in the OFC, which is regarded as a critical region for pain adjustment. Pain is one of the common symptoms of CAI, and approximately 60% of the patients report pain symptoms (Adal et al., 2020). Other studies have also proved the cerebral adaptation induced by chronic pain that OFC modulates the excessive excitement input under pain circumstances by compensatory inhibition (Li et al., 2020). In pain studies like chronic shoulder pain and primary dysmenorrhea, deceased regional cerebral activity was observed in the OFC (Jin et al., 2017; Li et al., 2020). In an arterial spin labeling study, abnormal cerebral blood flow was detected in the right middle frontal orbital gyrus in migraine sufferers (Zhang D. et al., 2021). Furthermore, the OFC serves a pivotal function in emotion modulation (Chen et al., 2021). According to previous studies, anxiety-related diseases show deactivation in the OFC. For example, the nodal parameters of OFC showed a negative correlation with Hamilton Anxiety Rating Scale, and the major depressive disorder and bipolar disorder patients had decreased FC in OFC (Chen et al., 2018; Wu et al., 2021). In a health-related quality of life study, the CAI patients demonstrated higher anxiety and depression score than the uninjured control group

(Kosik et al., 2020). Hence, we suggest that the depressed activity in OFC may be a biomarker of CAI patients with anxiety and depression symptoms.

The FC analysis was conducted to investigate the time series correlation analysis between ROI and the whole brain. The results demonstrated an increased FC between the right precentral gyrus and the right postcentral gyrus. In other words, the patients had enhanced communication within the sensorimotor network, which further supported the compensatory hypothesis. While decreased FC was observed between the right precentral gyrus and ACC, and the right middle frontal gyrus and left middle temporal gyrus. These diminished interhemispheric connections implicated that the CAI patients had deficits in functional synergy in distant brain regions. The ACC serves an essential role in guiding behaviors, coordinating and integrating information in motor selection and preparation, motivation response and evaluation (Zeuner et al., 2016). As a previous study reported, the ACC might modulate the supplementary motor area during performing unilateral motor tasks (Cordani et al., 2020). The middle temporal gyrus is a region responsible for processing visual signals like distance recognition and action perception (Conboy et al., 2021; Kang et al., 2021). It is known that the appropriate neuromuscular control depends on the apropos integration of the somatosensory, visual, and vestibular system (Peterka, 2002; Terada et al., 2020). A recent study revealed a low correlation between motor network and middle temporal cortex in individuals with chronic musculoskeletal impairment, which was consistent with our results. It might result from the long-term disuse of the affected limb (Conboy et al., 2021). In addition, the IPL is a part of the “pain matrix” responsible for pain processing, including discriminating and sensing pain and directing attention to deleterious stimulation and high sensory integration (Nakata et al., 2008; Silvestro et al., 2021; Tang et al., 2021). It further supported the idea that the neuroplasticity related to pain processing occurred in the CAI patient, likely presenting as an inhibitory neural correlation between the pain-related regions, the OFC and the IPL.

There were several limitations in this study. First, despite the sample size being relatively large among the cerebral research of CAI, it is still limited in fMRI studies, which somewhat limited the statistical validity. Secondly, although we collected scale scores related to clinical symptoms of the disease and ankle functions, we lacked behavioral or psychological data of patients. Patients’ emotional condition assessment should be collected to explore what role pain-related anxiety or depression plays in CAI.

## Conclusion

In the current study, we investigated the alteration of inherent cerebral functional activity in CAI patients. The results suggested the aberrant changes in the sensorimotor network and pain matrix among the CAI patients, which may be involved with compensatory neuromuscular strategy, pain processing, and psychological abnormality. These findings may provide insights into the pathophysiological mechanisms of CAI and facilitate the intervention and management improvement of this disease.

## Data Availability Statement

The original contributions presented in the study are included in the article/supplementary material, further inquiries can be directed to the corresponding authors.

## Ethics Statement

The studies involving human participants were reviewed and approved by the Huashan Hospital Institutional Review Board. The patients/participants provided their written informed consent to participate in this study.

## Author Contributions

HL and XW contributed to the conception and design of the study. YS wrote the first draft of the manuscript. WW and LY critically revised the manuscript. YW performed the data acquisition and analysis. CY performed the clinical assessment. YY, FW, JW, and YD revised sections of the manuscript. All authors contributed to manuscript revision and approved the submitted version.

## Conflict of Interest

The authors declare that the research was conducted in the absence of any commercial or financial relationships that could be construed as a potential conflict of interest.

## Publisher’s Note

All claims expressed in this article are solely those of the authors and do not necessarily represent those of their affiliated organizations, or those of the publisher, the editors and the reviewers. Any product that may be evaluated in this article, or claim that may be made by its manufacturer, is not guaranteed or endorsed by the publisher.
